# Interactions of the microbiome with pharmacological and non-pharmacological approaches for the management of ageing-related musculoskeletal diseases

**DOI:** 10.1177/1759720X211009018

**Published:** 2021-04-20

**Authors:** Maria Papageorgiou, Emmanuel Biver

**Affiliations:** Division of Bone Diseases, Geneva University Hospitals and Faculty of Medicine, University of Geneva, Geneva, Switzerland; Division of Bone Diseases, Geneva University Hospitals and Faculty of Medicine, University of Geneva, Rue Gabrielle Perret Gentil 4, Geneva 1205, Switzerland

**Keywords:** ageing, bone health, microbiome, osteoarthritis, osteoporosis, prebiotics, probiotics, sarcopenia

## Abstract

Despite major progress in the understanding of the pathophysiology and therapeutic options for common ageing-related musculoskeletal conditions (i.e. osteoporosis and associated fractures, sarcopenia and osteoarthritis), there is still a considerable proportion of patients who respond sub optimally to available treatments or experience adverse effects. Emerging microbiome research suggests that perturbations in microbial composition, functional and metabolic capacity (i.e. dysbiosis) are associated with intestinal and extra-intestinal disorders including musculoskeletal diseases. Besides its contributions to disease pathogenesis, the role of the microbiome is further extended to shaping individuals’ responses to disease therapeutics (i.e. pharmacomicrobiomics). In this review, we focus on the reciprocal interactions between the microbiome and therapeutics for osteoporosis, sarcopenia and osteoarthritis. Specifically, we identify the effects of therapeutics on microbiome’s configurations, functions and metabolic output, intestinal integrity and immune function, but also the effects of the microbiome on the metabolism of these therapeutics, which in turn, may influence their bioavailability, efficacy and side-effect profile contributing to variable treatment responses in clinical practice. We further discuss emerging strategies for microbiota manipulation as preventive or therapeutic (alone or complementary to available treatments) approaches for improving outcomes of musculoskeletal health and disease.

## Introduction

Chronic musculoskeletal conditions including osteoporosis and associated fractures, sarcopenia and osteoarthritis present major threats to healthy ageing.^1,2^ These conditions are commonly characterized by pain and reduced physical function and lead to significant disability, functional and mental health declines, and increased mortality.^1,2^ Their management includes non-pharmacological interventions (e.g. an active lifestyle, adequate intakes of dietary protein, calcium and vitamin D or weight management) and/or pharmacological interventions (e.g. non-disease-specific drugs that ameliorate pain or more specific ones targeting the pathophysiology of musculoskeletal diseases).^3–6^ Although continuous advances in these treatments have undoubtedly resulted in significant improvements in clinical parameters and quality of life for patients, treatment responses among patients are highly variable.^3,7–9^ Indeed, while some patients tolerate and respond well to treatment, some others gain little or no benefit, and/or experience adverse effects. Understanding the factors that contribute to variability in treatment responses is critical for providing safe and effective therapies, while meeting patients’ expectations and reducing wasteful health spending.

The microbiome, the diverse ecosystem of bacteria, fungi, archaea and viruses and their genetic material is now identified as an integral ‘human organ’.^10^ The microbiome is dynamic throughout the lifespan and varies greatly within and between individuals as a result of intrinsic and extrinsic factors.^10–12^ Most microbes live symbiotically with the human host and promote health through their involvement in a vast array of functions ranging from maintaining intestinal barrier function and nutrient fermentation to endocrine and immune functions. Conversely, perturbations in microbiota composition, functional and metabolic capacity, collectively termed dysbiosis, have been associated with intestinal and extra-intestinal disorders including musculoskeletal diseases.^11–17^ Besides its contributions to disease pathogenesis, the microbiome has an emerging role in disease therapeutics. This realization led to the development of ‘pharmacomicrobiomics’, a new discipline which investigates the interactions between drugs and the microbiome and how these affect clinical responses to drugs.^18–20^ This field has great potential to advance microbiome-related precision medicine approaches including strategies that predict response to therapies and interventions that reshape the microbiome to enhance treatment responses.

**Figure 1.**
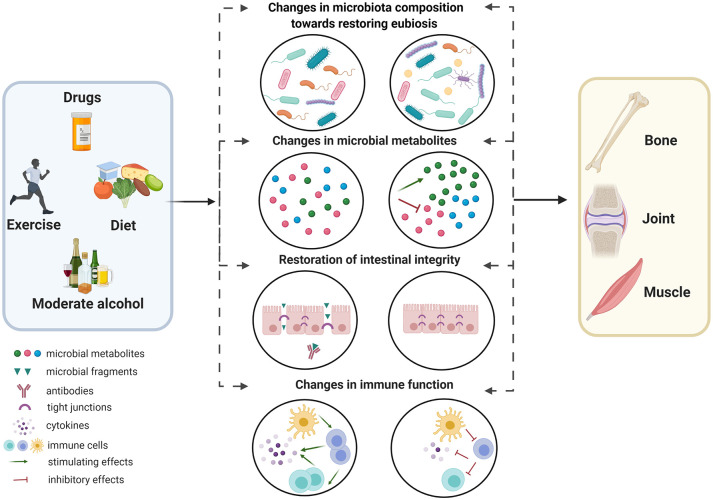
A summary of microbiota-related mechanisms through which therapeutics (drugs, nutraceuticals, and lifestyle changes) may positively affect outcomes related to bone, muscle and joint health and disease. These mechanisms include (a) modifications of microbiota composition towards restoring eubiosis, (b) changes in the production of microbial metabolites (i.e. increases in metabolites with potential health benefits such short-chain fatty acids, and reduction in microbial metabolites associated with disease), (c) restoration of intestinal integrity (promotion of tight junction of barrier function and inhibition of transfer of microbial fragments into the intestinal mucosal) and (d) regulation of the immune system (reduced activation of immune cells and cytokine production). Dashed arrows indicate that changes in one of these mechanisms may also have indirect effects on the others. Created with BioRender.com.

**Figure 2.**
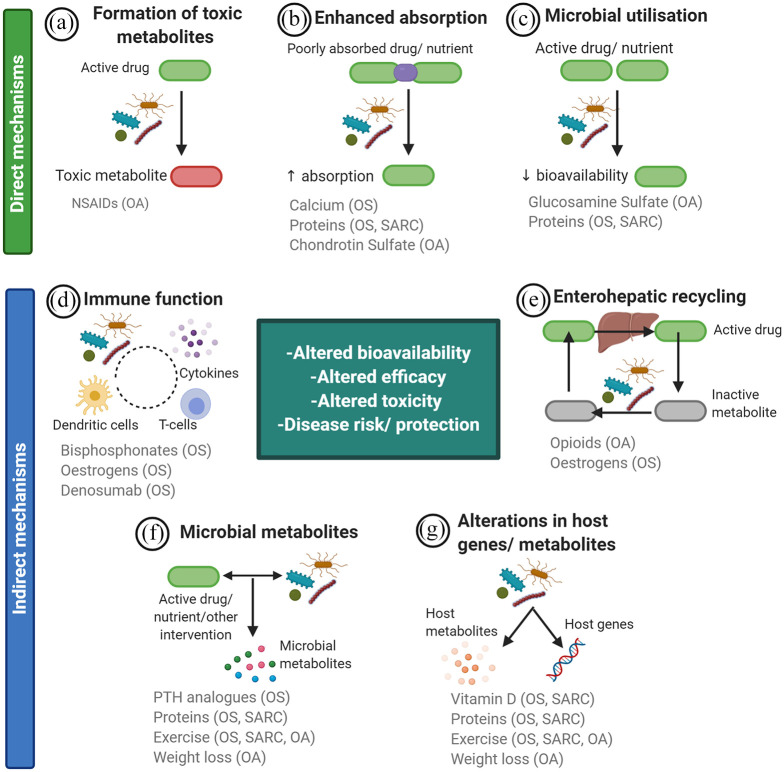
A summary of potential mechanisms by which the microbiota influences the bioavailability, efficacy and toxicity of therapeutics for ageing-related musculoskeletal diseases [osteoporosis (OS), sarcopenia (SARC), osteoarthritis (OA)], but also individuals’ susceptibility to disease. Direct mechanisms (a–c) include formation of toxic metabolites, release/metabolism of otherwise poorly absorbed nutraceuticals/nutrients (and thus, enhanced absorption) and utilization of the therapeutic compounds by microbiota with subsequent reductions in their amount available for absorption. Indirect mechanisms (d–g) include regulation of immune responses, microbial participation in enterohepatic recycling, production of microbial metabolites and altered production of host metabolites or host gene expression. Created with BioRender.com.

In this review, we focus on the complex interactions of the microbiome with pharmacological and non-pharmacological approaches for managing common ageing-related musculoskeletal diseases, namely osteoporosis, sarcopenia and osteoarthritis. Specifically, we identify (a) the effects of available treatments on microbiota composition and associated metabolites, intestinal integrity and immune function (Figure 1), and also (b) the effects of the microbiome on the metabolism of drugs/nutrients, which may influence their efficacy and side-effect profile and contribute to variable treatment responses in clinical practice (Figure 2). We further detail whether these latter effects are mediated *via* direct (e.g. utilization or biotransformation of drugs/nutrients or their metabolites into products with altered properties) or indirect (e.g. modulation of host immune function or host pathways for drug/nutrient metabolism) mechanisms. In the second part of this review, based on clinical data in adults, we discuss emerging approaches for the manipulation of microbiome to improve clinical parameters related to musculoskeletal health and disease, and/or treatment efficacy and side-effect profiles.

## Interactions between the microbiome and treatments for musculoskeletal disorders

### Treatments that control pain and/or inflammation

#### Acetaminophen

Acetaminophen’s metabolism in the liver gives rise predominantly to two inactive, non-toxic compounds (acetaminophen sulphate and acetaminophen glucuronide), and to minor amounts of N‑acetyl-p‑benzoquinone imine, which has been linked to hepatotoxicity.^18^ By using a metabolomics approach, Clayton *et al*.^21^ demonstrated that individuals with higher urinary levels of p-cresol (a microbial metabolite arising from amino acid fermentation) prior to acetaminophen administration, had a lower post-treatment urinary ratio of acetaminophen sulphate to acetaminophen glucuronide. Acetaminophen and p-cresol compete for sulphation by the cytosolic sulphotransferase, therefore, the more p-cresol produced by the gut bacteria, the less acetaminophen is sulphated and detoxified, shifting the metabolism of acetaminophen towards the pathway that leads to N‑acetyl-p‑benzoquinone imine production. Conceivably, intra-, and inter-individual variations in the diet (i.e. substrate availability) and gut microbial capacity to form p-cresol can contribute to within- and between- individual discrepancies in acetaminophen metabolism and hepatotoxicity.^22,23^

#### Opioids

Opioids are potent analgesics; their use is, however, limited by side effects including addiction, gastrointestinal symptoms and sedation.^24^ Animal and human studies suggest that opioid use compromises gut homeostasis contributing to impaired immune responses and susceptibility to infectious diseases and gut-origin sepsis. Specifically, treatment with opioids has been associated with reduced intestinal motility, inhibition of protective mechanisms for the gut epithelium (e.g. mucus, bicarbonate release), altered gut microflora composition (e.g. growth of Gram-positive pathogens and reduction in bacteria that metabolize bile acids) and loss of gut barrier function, allowing bacterial translocation.^24,25^ Such microbial dysbiosis might also influence the metabolism of these drugs. Morphine is deconjugated in the liver to morphine-3-glucuronide that exerts no analgesic effects.^24^ In the gut, this metabolite can be reconverted to morphine by microbial β-glucuronidases and reabsorbed into the circulation. An increased morphine-3-glucuronide-to-morphine ratio in the gut and reductions in bacteria that encode β-glucuronidases following morphine treatment suggest reduced enterohepatic recycling of this drug and potentially altered analgesic effects.^26^

#### Nonsteroidal anti-inflammatory drugs (NSAIDs)

Despite their efficacy in ameliorating pain and inflammation in osteoarthritis and inflammatory arthritis, chronic non-steroidal anti-inflammatory drug (NSAID) use is concerning due to associated upper and lower gastrointestinal (GI) side effects in a substantial proportion of patients. The gut microbiome has been lately implicated in the development of NSAID-induced enteropathy.^27,28^ Evidence from animal work indicates that germ-free (GF) animals (devoid in microbiota) are protected against NSAID enteropathy, whereas the re-introduction of microbiota leads to intestinal damage.^27^ Mechanistically, the inhibition of prostaglandin synthesis by NSAIDs increases the susceptibility of the intestinal mucosa to injury and impairs its repair capacity.^28^ In parallel, NSAIDs have been associated with marked alterations in the variety and abundance of intestinal microbiota, which contribute to the development of enteropathy by compromising mucosal integrity and allowing the penetration of pathogens, but also by interfering with the metabolism of these drugs.^27–29^ Specifically, microbial β-glucuronidases deconjugate the metabolites of several NSAIDs and release aglycones which can induce gut toxicity.^18^ Administration of β‑glucuronidase inhibitors to animals treated with NSAIDs was shown to reduce mucosal injury and enteropathy compared to animals not receiving the inhibitors.^30^ The feasibility of this strategy to reduce NSAID enteropathy remains to be explored in humans.

To reduce the upper GI side effects of NSAIDs, NSAID users are often co-prescribed proton pump inhibitors (PPIs); these agents may, however, potentiate the NSAID-induced enteropathy by exacerbating dysbiosis.^27,31^ In animal experiments, PPIs worsened the intestinal damage and bleeding caused by NSAIDs.^27^ These results were attributed to pronounced alterations in gut microbiota composition (i.e. reductions in *Actinobacteria* and *Bifidobacteria* spp.), while daily supply of bifidobacteria reversed dysbiosis and prevented NSAID-induced ulceration.^27^ In the same study, GF mice colonized with bacteria from healthy animals experienced mild intestinal injury following NSAID treatment, whereas colonization of GF mice with bacteria from PPI-treated rats resulted in severe NSAID-induced intestinal damage.^27^ In humans, PPI users display decreased alpha diversity (i.e. reduced number of microbial species) and changes in 20% of bacterial taxa compared with non-users,^32^ while an increased abundance of *Bacteroides* and *Erysipelotrichaceae* spp. can discriminate the combined use of NSAIDs and PPIs from NSAID use alone.^29^

### Treatments that target bone remodelling

#### Bisphosphonates

Few studies have explored the bisphosphonates–microbiome interactions. *In vitro* studies have demonstrated that bisphosphonates possess antimicrobial properties,^33^ nevertheless, it is unclear if such actions are exerted in humans or whether/how these are related to the clinical efficacy or toxicity of these agents. For example, patients with bisphosphonate-induced jaw osteonecrosis, a very rare but severe side effect of bisphosphonate use, have been reported to have different oral microbiome and increased pathogenic bacteria than non-affected patients.^34^ The microbiome may further influence host immune responses to inflammation, bacterial infections and wound healing, thus, predisposing or protecting the host to/from this side effect.^35,36^

#### Oestrogens and selective oestrogen-receptor modulators

Mounting evidence suggests a reciprocal relationship between oestrogens and the microbiome with implications for osteoporosis treatment with hormonal replacement therapy and selective oestrogen-receptor modulator (SERMs). A series of experiments that compared conventional with GF female mice following oestrogen depletion using the gonadotropin-releasing hormone (GnRH) agonist, leuprolide, to mimic postmenopausal status, revealed that the gut microbiota has a pivotal role in sex-deficiency-induced osteoporosis.^37^ While conventional mice lost trabecular bone, the GF mice were protected from it. The favourable bone phenotypes in the GF animals were attributed to their failure to increase bone marrow CD4^+^ cells and T-helper 17 (Th17) cells (an osteoclastogenic sub-population of CD4^+^ cells), pro-osteoclastogenic cytokines (tumour necrosis factor alpha, TNF-α; receptor activator of nuclear factor kappa-B ligand, RANKL; and interleukin 17, IL-17) and osteoclast numbers, as well as to their ability to maintain barrier function.^37^ Convincingly, these phenotypes were reversed following colonization of GF animals with microflora from conventional mice. Further reinforcing the idea of an oestrogen–microbiota interplay, treatment with oestrogens in animal models has been associated with significantly higher microbial diversity,^38^ reduced growth of lipopolysaccharides (LPS)-producing Gram-negative bacteria,^39^ changes in bacterial activity,^40^ tightening of gut permeability and decreased inflammation induced by high-fat feeding.^39^ Interestingly, in postmenopausal women or animal models of human menopause, treatment with antibiotics,^41,42^ probiotics^37,43–46^ and prebiotics^47,48^ can blunt the bone loss induced by sex-steroid depletion by several mechanisms, including strengthening the gut barrier integrity and dampening intestinal and systemic inflammation. Although most of these findings originate from animal studies, and hence, require replication in humans, they suggest a feedback loop between oestrogens and the gut microbiome that influences the immune system. They also raise the possibility that oestrogen-based therapies might be improved by gut microbiota modulation that may work independently or synergistically to ameliorate inflammation and bone loss.

Conversely, the gut microbiome can metabolize oestrogens, hence, regulating their circulating levels. Some bacteria possess genes (collectively termed the estrobolome) that encode β-glucuronidases, which deconjugate oestrogens into their biologically active forms.^40^ The deconjugated oestrogens can be reabsorbed into the circulation and bind to their receptors in peripheral tissues to elicit their biological effects. In postmenopausal women who were not taking oestrogens or antibiotics, the ratio of oestrogen metabolites to their precursors^49^ and systemic oestrogen concentrations^50^ were shown to be proportional to their gut microbial diversity and/or deconjugation potential. Although the associations between the microbiome, oestrogen metabolism and bone outcomes in women with/at risk of osteoporosis on synthetic/exogenous oestrogens remain to be elucidated, the evidence of extensive oestrogen metabolism in the gut raises a number of scenarios relevant to oestrogen therapies. Differences in gut microbial composition and glucoronidase activity may contribute to the variability in oestrogen-based therapies responses.^9^ Furthermore, oestrogen-based therapies may induce changes in bacterial composition and microbial-mediated deconjugation process,^40^ with implications for the half-life, efficacy, and safety of these agents and potential benefits to be gained from their co-administration with specific nutrients or bacteria.

#### Denosumab

Denosumab is a humanised monoclonal antibody that binds with high affinity to RANKL to block the development and function of osteoclasts, and thereby, it reduces bone resorption.^3^ Although no study has explored the effects of denosumab on the microbiome of animals/individuals with osteoporosis, a recent animal study using an experimental model of colitis demonstrated that mice treated with denosumab experienced altered alpha and beta diversity (differences in microbial composition between samples) of faecal microflora and downregulated inflammatory responses (pro-inflammatory cytokines IL-6, IL-1β, and TNF-α) in the colonic mucosa.^51^ Further indirect evidence from supplementation studies in relevant animal models (e.g. sex steroid deficiency,^37,43,46^ inflammation^52^) suggest that treatment with probiotics reduces bone resorption and improves bone mineral density (BMD), while mechanistically, these outcomes may be partially mediated by alterations in the RANKL/RANK/OPG (osteoprotegerin) signalling pathway (e.g. downregulation of RANKL and pro-inflammatory cytokines and/or upregulation of OPG and anti-inflammatory factors). Based on these findings which require further investigations in animal and human trials, it can be speculated that denosumab may have microbiome- and immune-modulating properties, while manipulations of the microbiome may influence musculoskeletal outcomes through tipping the RANKL–OPG balance.

#### Parathyroid hormone (PTH) analogues (teriparatide and abaloparatide)

Although no previous study has explored the interactions between the available parathyroid hormone (PTH) analogues, teriparatide and abaloparatide, and the microbiome in humans, two recent studies implicate the gut microbiome in PTH-mediated bone formation^53^ and bone resorption.^54^ The microbiome *via* butyrate production [a short-chain fatty acid (SCFA) produced in response to microbiota-dependent fibre fermentation, with overall beneficial effects for the host] appears to be essential for PTH anabolic actions. In particular, treatment with intermittent PTH increased trabecular bone-volume fraction, trabecular thickness and number in mice with replete microbiota (conventionally raised animals), but these effects were not observed in GF and antibiotic-treated mice with depleted microbiota, and thus, deficient in microbiota-derived butyrate.^53^ There were no differences between groups in parameters of cortical bone, suggesting that the microbiota is involved in the mechanism through which PTH increases trabecular, but not cortical bone mass. The authors further demonstrated that butyrate supplementation in mice treated with antibiotics reversed the ability of PTH to promote anabolism. Butyrate increased Tregs in the intestine and bone marrow and enabled PTH to differentiate naïve CD4^+^ T cells into Tregs, a lineage of T cells which induce CD8^+^ T cells to release Wnt10b. Wnt10b is a powerful activator of Wnt signalling in stromal cells and osteoblasts, and thus, it stimulates osteoblast recruitment and lifecycle, and ultimately, bone formation. Butyrate’s effects on enhancing the anabolic effects of PTH were mediated through binding to its receptor (GPR43) on dendritic cells, but also by interacting with T cells *via* a GPR43-independent pathway.^53^ The translation of these data to humans and their significance for treatment with PTH analogues await future study. Given that responders to teriparatide treatment experience BMD gains of different magnitudes, while a subgroup of patients show no BMD improvement after this treatment,^7^ it would be important to explore whether these variable responses are at least partially explained by inter-patient disparities in the microbiome and/or SCFA/butyrate availability. Future studies should also investigate whether the addition of probiotics, prebiotics or SCFAs could make patients’ microbiomes, especially those microflorae which are less abundant in butyrate bacterial taxa, more receptive to the anabolic effects of PTH analogues.

### Calcium and vitamin D

#### Calcium

Given the importance of calcium for bone structure and homeostasis, an adequate calcium intake is recommended for osteoporosis prevention and treatment, while an adequate intake of dietary calcium may also be beneficial for weight management and glycaemic control.^55^ Several dietary nutrients including calcium are digested and released by intestinal bacteria, while microbiota manipulation with prebiotics (for a detailed description see section ‘Intervention studies with prebiotics’ and Table 1) improves calcium bioavailability through several possible mechanisms.^12^ First, the transformation of prebiotics to SCFAs lowers the pH in the intestinal lumen; such changes are alleged to hinder the formation of calcium–phytate/oxalate complexes, thereby increasing the amount of available calcium for absorption. Second, the ingestion of prebiotics has been associated with increases in cell density, intestinal crypt depth, and blood flow, which may increase intestinal surface area and enhance calcium absorption.^56^ Third, prebiotics (possibly through resulting SCFAs) may upregulate the expression of intracellular calcium transporters.^48^ Increases in calcium absorption, reduce PTH and may have downstream effects on several tissues including bone (i.e. reduction in bone resorption), adipose tissue (e.g. activation of lipogenesis and suppression of lipolysis, formation of calcium-fatty acid soaps), and muscle (e.g. increased glucose uptake and insulin sensitivity).^55^

**Table 1.** A summary of clinical studies evaluating the effects of prebiotics on outcomes related to musculoskeletal health and disease in adults.

Study	Population (age, number)	Study Design	Intervention(s)	Control	Outcomes	Main findings
Calcium balance, BTMs and BMD						
Van den Heuvel *et al*.^57^	Postmenopausal women (⩾5 years since menopause), age: 56–64 years, *n* = 12	Placebo-controlled, randomized, crossover design, double isotope method; 19-day washout period, D: 9 days	5 or 10 g/day of lactulose dissolved in 100 ml of water with benzoic acid	Aspartame dissolved in 100 ml of water with benzoic acid (0 g lactulose)	Ca absorption	Dose-response ↑ in Ca absorption with lactulose supplementation; Ca absorption with 10 g lactulose was significantly higher (*versus* control), but there were no differences between 5 g and 10 g of lactulose; NS differences in Ca excretion between treatments
van den Heuvel *et al*.^58^	Postmenopausal women (⩾5 years since menopause), age 55–65 years, *n* = 12	Placebo-controlled, double-blind, randomized, crossover design, double isotope method; D: 9 days	20 g/day of trans-GOS in 200 ml of yogurt (gradual dose increase from 10 g/day to 20 g/day over 5 days)	20 g sucrose in 200 ml of yogurt	Ca absorption	↑ Ca absorption with 20 g of GOS (*versus* control); NS differences in Ca excretion between treatments
Tahiri *et al*.^59^	Postmenopausal women (>2 years since menopause) without HRT, age 50–70 years, *n* = 12	Double-blind, randomized crossover, design, single isotope method, 3-week washout period, D: 5 weeks	10 g/day short-chain FOS	Sucrose	Ca absorption BTMs	NS effect of short-chain FOS on Ca absorption (*versus* control); NS differences in Ca excretion between treatments BTMs: NS changes within or between groups
Kim *et al*.^60^	Postmenopausal women (~12 years since menopause), mean age 60 years, *n* = 26	Randomized, double-blind parallel design, D: 3 months	8 g/d of FOS	Maltodextrin–sucrose	Ca absorption BTMs BMD	↑ Ca absorption after FOS (*versus* control) with no differences between groups in urinary calcium excretion BTMs: ↓ serum ALP after FOS (*versus* control) BMD: NS difference in spine or hip BMD after FOS
Abrams *et al*.^61^	Women and men, age 18–27 years, *n* = 13	Kinetic modelling study conducted in responders to intervention (>3% ↑ in Ca absorption), double isotope method, D: 8 weeks	Participants had diets with Ca 800–1000 mg/d and received 8 g/d ITF mixed with 120 ml Ca + vitamin-D-fortified orange juice	None	Ca absorption	↑ Ca absorption in the colon (*versus* baseline) in responders Colonic absorption was defined as absorption that occurred >7 h after oral dosing
Holloway *et al*.^62^	Postmenopausal women (⩾10 years since menopause), mean age: 72 y, *n* = 15	Double-blind, placebo-controlled, randomised, crossover design, DI, 6-week washout period, double isotope method, D: 6 weeks	IN + oligofructose 10 g/d (SYN1)	Maltodextrin	Ca absorption BTMs	↑ Ca absorption in SYN1 group (*versus* baseline and *versus* control), NS changes in the control group BTMs: ↑ uDPD and ↑ uOC (*versus* baseline) in SYN1 group only
Adolphi *et al*.^63^	Postmenopausal women (⩾10 years since menopause), age 48–67 years, *n* = 85	Double-blind parallel design; matched for age, time after menopause, BMI and dietary calcium intake, D: 2 weeks	f-milk with extra Ca (f-milk + Ca) or f-milk with extra Ca, IN-type fructans and caseinphosphopeptides (f-milk + Ca + ITF + CPP)	f-milk	Ca absorption BTMs	↑uCa during night-time in the f-milk + Ca + ITF + CPP BTMs: ↓nocturnal urinary excretion of DPD
Slevin *et al*.^64^	Postmenopausal women, age 45–75 years, *n* = 300	Double-blind, randomized, parallel design, D: 24 months	800 mg/day Ca (Ca) or 800 mg/day Ca + 3.6 g/day short-chain FOS (CaFOS)	9 g/day of maltodextrin	BTMs BMD	BTMs: Greater ↓ in CTX in the CaFOS and Ca groups (*versus* control) at 12 months; greater ↓in OC in the CaFOS group (*versus* control) at 24 months NS differences in BMD changes after Ca or CaFOS *versus* control
Tu *et al*.^65^	Women and men with osteoporosis, mean age ⩾64 years, *n* = 40	Double-blind, randomized, parallel design, included those with and without fracture, D = 6 months	Kefir-fermented milk (1600 mg) + calcium bicarbonate (1500 mg/day)	Calcium bicarbonate (1500 mg/day)	BTMs BMD	NS differences in serum calcium between treatment groups BTMs: ↑ CTX and ↑ OC in the treatment group *versus* control at 6 months; the impact of kefir treatment on total hip and femoral neck BMD was associated with OC levels at baseline NS difference in spine or hip BMD after 6 months between treatment groups
Jakeman *et al*.^66^	Postmenopausal women (4+ years after menopause), age 40–78 years, *n* = 12	Placebo-controlled, randomized, crossover design, single isotope method, 50-day washout period, D: 50 days	10 or 20 g/day SCF (Promitor10)	0 g/day SCF	Ca absorption BTMs	A dose-dependent ↑ whole-body Ca retention with 10 g/d and 20 g/d SCF BTMs: bone ALP ↑ with 20 g/day SCF (*versus* control), NS changes in sOC or uNTX
Kruger *et al*.^67^	Premenopausal women, mean age 41 years, *n* = 136 Postmenopausal women, mean age 59 years, *n* = 121	Randomized, parallel design, D: 12 weeks	Ca (1000–1200 mg/d) and fort-milk (15 ug/d) + FOS (4 g/day) (fort-milk + FOS)	Regular milk (500 mg Ca/day)	BTMs	Postmenopausal women: greater ↓ in CTX and PTH in the fort-milk + FOS group (*versus* control) at 12 weeks; no difference in premenopausal women
Sarcopenia/frailty-related outcomes						
Buigues *et al*.^68^	Older individuals, age ⩾65 years, *n* = 50	Placebo-controlled, randomized, double blind design, D = 13 weeks	IN + FOS	Maltodextrin	Physical performance Activities of daily living	↓ self-reported exhaustion and ↑ handgrip strength in the IN + FOS group (*versus* control); NS changes in Barthel index, BMI or frailty rate after IN + FOS (*versus* control)
Theou *et al*.^69^	Ambulatory elderly living in nursing homes, age ⩾65 years, *n* = 50	Placebo-controlled, randomized, double blind design, D = 13 weeks	IN + FOS	Maltodextrin	Frailty	Frailty index ↑ in the placebo group, whereas it ↓ in the intervention group

↑, increased; ↓, decreased; BAP, bone alkaline phosphatase; BMD, bone mineral density; BMI, body mass index; BTMs, bone turnover markers; Ca, calcium; CTX, C-telopeptide of type 1 collagen; D, duration; DPD, deoxypyridinoline cross-links; f-milk, fermented milk; fort-milk, vitamin-D-fortified milk; FOS, fructo-oligosaccharides; GOS, galacto-oligosaccharides; HRT, hormonal replacement therapy; IN, inulin; ITF, inulin-type fructans; *n*, number; NS, non-significant; NTX, N-terminal telopeptide of type I collagen; OC, osteocalcin; PTH, parathyroid hormone; s, serum; SCF, soluble corn fibres; u, urinary; SYN1, Synergy1®.

The beneficial effect of calcium ingestion on metabolic health, and possibly skeletal health, could be associated with modifications of intestinal microbiota and integrity.^55^ High-calcium diets or calcium supplementation in animal models of diet-induced obesity (*versus* control animals or animals fed low-calcium diets) promote the proliferation of potentially beneficial bacteria and SCFA production, attenuate the damage of intestinal mucosal and LPS translocation, prevent endotoxemia and regulate tight junction gene expression.^55,70–72^ Future preclinical and clinical research is required to investigate the calcium–microbiome interactions in relation to musculoskeletal and metabolic outcomes in dysbiotic conditions (ageing, menopause, obesity).

#### Vitamin D

Vitamin D has well-established roles in calcium homeostasis, bone growth/maintenance and muscle function.^4,73,74^ More recently, the discovery that the vitamin D receptor (VDR) and the vitamin D activating enzyme 1-α-hydroxylase (CYP27B1), are present in bone and kidneys, but also on cells in the intestine, muscle, pancreas, prostate and immune system, has unravelled numerous extra-skeletal effects of vitamin D, including immunomodulation, maintenance of intestinal homeostasis and gut microbiota eubiosis.^75,76^ Dysbiotic gut microbiota profiles with increased abundance of the phyla Bacteroidetes and associated taxa have been reported in mice fed diets low in vitamin D (*versus* mice fed diets high in vitamin D) and VDR or CYP27B1 knockout mice (*versus* wild-type mice).^77^ Additional investigations provided evidence that vitamin D deficiency compromises the mucosal barrier integrity (by disrupting junction proteins) and antimicrobial functions at epithelial surfaces.^75,78^ Such changes may allow opportunistic bacteria to outperform commensal bacteria, resulting in the upregulation or downregulation of immune responses. These findings are consistent with findings from human studies supporting that gut microbiota composition, circulating levels of LPS and inflammatory markers differ according to vitamin D intakes or vitamin D levels.^79,80^ Furthermore, vitamin D supplementation may reinstate a healthier gut microbiome profile and lessen inflammation,^80–82^ with these effects shown to be dose dependent ^80^ and more pronounced in the upper GI tract.^81^

The gut microbiome can also regulate vitamin D metabolism, and potentially, status and function. This is supported by preclinical data demonstrating that some intestinal bacteria possess hydroxylases that activate vitamin D.^83^ Well-known regulators of vitamin D levels may be involved in the relationship between vitamin D metabolism and microbiota.^84^ A recent study demonstrated that GF mice exhibit impairments in vitamin D metabolism (high FGF23 and low 1,25-dihydroxvitamin D levels), which are progressively restored following reductions in FGF23; while conventionalization of GF mice induces inflammation that inhibits FGF23, resulting in increases in 1,25-dihydroxvitamin D and calcium levels.^84^ In addition to these findings, administration of probiotics in animals or humans has been shown to enhance vitamin D levels and upregulate VDR expression and activity in the host; while probiotic protection against bacterial infection and gut inflammation appear to be dependent on the VDR signalling pathway.^85–87^

### Nutraceuticals for the management of osteoarthritis

Chondroitin sulphate and glucosamine sulphate are slow-acting nutraceuticals (i.e. foods or parts of a food that provide medical or health benefits) with widespread use among patients with osteoarthritis.^88,89^ Studies investigating their efficacy on disease-related outcomes have yielded mixed results, with a recent meta-analysis suggesting improvements in pain intensity and physical function post-supplementation in patients with hip or knee osteoarthritis.^88,90^ Several lines of evidence suggest that the gut microbiome plays a significant role in the bioavailability of both supplements. Chondroitin is poorly absorbed in the small intestine and requires degradation to its component disaccharides by gut bacteria before it can be absorbed.^91^ In contrast to chondroitin, glucosamine is a monosaccharide largely utilized by gut bacteria, hence, the amount of the drug available for absorption is limited.^92^ Further, chondroitin degradation rates vary among individuals due to their distinct bacterial compositions, with this observation providing an explanation of inter-patient differences in the efficacy of equal doses of the drug.^93^

On the other hand, glucosamine sulphate and chondroitin sulphate may exert some of their joint protective effects though modifying gut bacterial growth and metabolic activity, but also *via* effects on gut mucosal immunity.^94^ A recent systematic review of animal and human studies concluded that chondroitin sulphate supplementation consistently increases the relative abundance of the genus Bacteroides, while there was insufficient evidence to draw conclusions on the effects of glucosamine sulphate on gut microbial composition.^94^ Interestingly, some species of the Bacteroides genus may use chondroitin as an energy source, thus, sparing other endogenous energy sources (e.g. intestinal mucins) and preventing gut inflammation.^94,95^ Similarly, some sulphate-reducing bacteria belonging to Bacteroides and able to cleave sulphate groups from chondroitin sulphate, appear to be involved in the production of anti-inflammatory molecules.^96^ Further indirect evidence suggests that since glucosamine and chondroitin are important constituents of the intestinal mucins, supplementation with these nutraceuticals may boost gut barrier function and intestinal immune responses.^97,98^

### Lifestyle changes

#### Protein intake

Adequate consumption of dietary protein is important for maintaining musculoskeletal integrity.^99^ The gut microbiome is implicated in protein metabolism and utilization. The intestinal bacteria have the capacity to break down undigested protein into amino acids, but also *de novo* synthesize essential amino acids from nitrogen sources.^100–102^ In the small intestine, microbial-derived amino acids can be taken up by the host and added to amino-acid plasma pools and body proteins. Interestingly, gut microbiota may enhance the amino-acid balance in individuals with low-protein intakes^102^ and improve the bioavailability of amino acids of significance for musculoskeletal health (e.g. leucine).^103,104^ In the large intestine, the amino acids resulting from bacterial protein degradation are mainly utilized by luminal bacteria or further catabolized by the microbiota to yield a range of metabolites [i.e. SCFA, branched-chain fatty acids (BCFAs), ammonia, hydrogen sulphide, indolic compounds, amines and polyamines] which exert positive or negative effects on microbial composition, intestinal homeostasis and other host functions.^100–102,105^ Thus, a healthy gut microbiota further contributes to host protein metabolism indirectly by controlling immune responses and inflammation, preventing insulin resistance, modulating host gene expression and maintaining gut barrier and mitochondrial function, whereas dysbiosis due to ageing or disease may negatively affect these parameters.^106^

On the other hand, dietary protein intakes influence the gut microbiota and host health through changes in microbiota composition and metabolic activities and host gene expression.^105,107–110^ These effects are complex and depend on the quantity of the protein, but also on protein sources, several dietary factors, and host characteristics. Indeed, increases in dietary protein have been associated with modest alterations in gut microbiota composition, unless these increases are accompanied by caloric and/or carbohydrate restrictions.^107–109^ High-protein diets consistently increase the amount of undigested protein reaching the large intestine causing a transition towards protein fermentation and production of relevant metabolites; however, the quantity and quality of this metabolic output may considerably vary according to various factors. For example, plant and animal proteins differentially affect metabolomes and gene expression in the rectal mucosa, with these effects possibly related to their different digestibility and amino-acid composition.^108^ High-protein, low-carbohydrate diets have been associated with increased production of potentially harmful metabolites (phenylacetic acid, N-nitroso compounds) and reduced production of beneficial metabolites (butyrate and phenolic acids),^109^ whereas supplementation of complex carbohydrates during high-protein diets lowers p-cresol, a microbial metabolite associated with disease.^111,112^ Further, since distinct bacterial species have different capacities to catabolize amino acids,^105^ the same protein intervention may have variable effects in individuals with distinct gut bacteria.

These findings are relevant to musculoskeletal health research and open new avenues for future work. Are the musculoskeletal benefits often seen with habitually high protein intakes and protein supplementation in populations at risk for musculoskeletal conditions^99,113–115^ mediated by gut microbiota? Is the absence of anabolic responses to protein or amino-acid supplementation reported in some studies^116–119^ related to the counteraction of positive protein effects by the negative impact of some amino-acid-derived metabolites or intestinal inflammation? If this is the case, can we develop interventions (e.g. protein–probiotic co-supplementation; high-protein, high-fibre diets) to reduce the negative effects associated with protein fermentation and maximize musculoskeletal benefits?

#### Physical activity

Having a high cardiorespiratory fitness^120,121^ or an active lifestyle^122^ and participation in sports^123,124^ or novel endurance exercise^125^ has been associated with largely positive modulations in gut microbiome such as enhanced diversity, proliferation of beneficial bacteria and elevated faecal SCFAs. Some of these effects seem to occur independently of diet, and fade upon return to a sedentary lifestyle.^125^ Further, indirect evidence supports positive or negative effects of exercise on gut transit time, inflammatory markers, immune function and intestinal barrier integrity depending on exercise modality, intensity, and duration.^126^ Notably, most of this evidence comes from preclinical studies and studies in athletes/physically active or sedentary healthy individuals, while the impact of exercise in dysbiotic conditions (e.g. obesity, diabetes, ageing), which predispose to musculoskeletal diseases, is understudied.^127^ To this end, animal studies suggest that exercise may partially reinstate gut dysbiosis and impairments in intestinal villi morphology induced by high-fat feeding.^128^ Lambert *et al*.^129^ also demonstrated interactions between exercise and diabetic status on gut microbial ecology in a mouse model of type 2 diabetes. In older individuals, two interventions involving aerobic exercise resulted in compositional changes in gut microbiota and parallel increases in cardiorespiratory fitness.^130,131^

The microbiome also has marked effects on exercise capacity and metabolic parameters and it has been recently proposed to contribute to the inter-individual variability in the responses to exercise training in humans.^132^ Evidence from animal studies suggest that GF and antibiotic-treated mice exhibit reduced endurance exercise capacity,^133–136^ skeletal muscle atrophy^137^ and impaired *ex vivo* skeletal muscle contractile function;^135^ whereas gut microbiota restoration through natural reseeding,^135^ SCFA supplementation^137^ or acetate infusion^136^ (another SCFA with potential benefits for the host) reverses these phenotypes. These gut microbiome effects on exercise responses are likely mediated by altering SCFA availability, substrate utilization and storage, oxidative stress, neural and immune functions or by interacting with mitochondria in energy production and inflammation.^132–134,138^ In humans, we have currently limited understanding on how distinct gut microbiomes affect responses to exercise. One such study demonstrated that the microbiotas of sedentary lean and obese individuals had different baseline compositions and responded differently to a 6-week endurance exercise intervention.^125^ Exercise increased SCFA-producing taxa and faecal SCFA concentrations in lean, but not obese, individuals, and these effects were independent of diet. These findings suggest that some individuals may be more responsive to a given exercise intervention than others and these discrepancies may be related to differences in their gut microbiome.

#### Weight loss

Weight loss appears to have differential effects on different aspects of musculoskeletal health. Weight loss of at least 10% of body weight is recommended for obese patients with osteoarthritis.^139^ Weight loss may also improve physical function in obese frail individuals;^140^ however, it has been associated with bone and muscle loss, impaired bone microstructure and elevated fracture risk, especially among elderly.^141,142^

In a bidirectional relationship, baseline microbiota composition (e.g. richness and certain types of bacteria) can predict responsiveness to weight-loss diets,^143^ and conversely, weight loss interventions involving dietary restrictions,^143–145^ bariatric surgery^143,145,146^ or direct microbiota modifications^143^ can affect microbial composition and associated metabolites. Nevertheless, the weight-loss–microbiome interactions have been rarely characterized in relation to musculoskeletal outcomes.^147^ A meta-analysis of 11 trials demonstrated that restrictive diets for weight loss were associated with lower total bacterial abundance and overall reductions of butyrate-producing bacteria (*Firmicutes*, *Lactobacillus* spp. and *Bifidobacterium* spp.), which were mostly related to macronutrient composition/deficiency. In the same meta-analysis, inconsistent results were observed for alpha diversity and compositional changes at phylum level.^143^ Besides alterations in microbiome composition, individual and total SCFA concentrations remain unaltered or significantly decreased, especially in the presence of low-carbohydrate/fibre intake, in response to weight-loss diets.^145^ Reductions in another gut microbial metabolite, namely trimethylamine N-oxide (TMAO), and its precursors (choline and L-carnitine) have been documented following dietary weight loss interventions; such changes may benefit cardiometabolic health, but, surprisingly, were associated with impairments in bone health among overweight/obese individuals.^147^

#### Smoking

Smoking is considered an important environmental risk factor in the pathogenesis of musculoskeletal diseases, while its avoidance is recommended as part of the management of patients with osteoporosis.^148,149^ Several human studies suggest that smoking alters microbial communities at different sites of the body (i.e. mouth, nose, lungs, upper and lower GI) sometimes towards a dysbiotic profile.^150–153^ A recent review that summarized the results of observational and interventional studies on the associations between intestinal microbiota and smoking suggested that overall, the intestinal microbiota of smokers are characterized by reduced microbial diversity, and abundance (phyla: Proteobacteria and Bacteroidetes, genera: Clostridium, Bacteroides and Prevotella) or scarcity (phyla: Actinobacteria and Firmicutes, genera: Bifidobacteria and Lactococcus) of certain phyla and genera.^150^ The effects of smoking of the microbiome are postulated to be due to direct contact with tobacco smoke, exposure to pathogenic micro-organisms and chemicals present in tobacco cigarettes, and/or tobacco metabolites, which, in turn, affect microbial populations *via* alterations in the GI microenvironment (i.e. oxygen, pH and acid), influences on the integrity of intestinal tight junctions and the composition of intestinal mucin, and immunosuppression.^150^ Such interactions might contribute to the development of intestinal and systemic diseases;^151^ nonetheless, the associations between smoking, dysbiosis and musculoskeletal diseases are unexplored. Smoking cessation has been associated with improved microbial diversity and favourable bacterial composition,^154^ raising the question whether the skeletal benefits seen after smoking cessation in osteoporotic patients are partially attributable to augmentations in dysbiosis.

#### Alcohol

Alcohol abuse and high alcohol intake have been shown to increase the risk for several diseases including osteoporosis and sarcopenia *via* different mechanisms,^155,156^ and the promotion of dysbiosis has been proposed as one of them.^157^ Heavy drinking has been associated with alterations in gut microbiota composition (e.g. growth of Proteobacteria and depletion of Bacteroidetes) and metabolites (reduced butyrate levels), barrier function disruption (increased LPS levels) and intestinal inflammation (increased levels of pro-inflammatory cytokines).^157,158^

Less clear are the effects of low-to-moderate alcohol consumption on musculoskeletal health, with some epidemiological studies suggesting reduced risk of osteoporosis in moderate alcohol drinkers.^156,159^ Besides the amount of ethanol, it is likely that the observed associations are influenced by other factors specific to the type of alcoholic drinks. Compared with distilled alcoholic beverages (e.g. spirit and liquors: ~40% alcohol by volume), fermented alcoholic drinks such as wine and beer are lower in alcohol content (beer: 2–8%; wine: typically ⩽14% alcohol by volume) and contain nutrients (i.e. polyphenols and fibres) with potential beneficial effects on the microbiome.^155^ For example, in a randomized cross-over trial, exclusive alcohol consumption (gin) over 20 days resulted in Bacteroides and Clostridium enrichment and scarcity of Prevotellaceae compared with baseline microbiota (no alcohol, no polyphenols) or in comparison with the microbiota changes seen during the 20-day periods of de-alcoholized red-wine consumption (polyphenols only) and red-wine consumption (alcohol and polyphenols).^160^ Furthermore, the consumption of de-alcoholized red wine and red wine resulted in growth of beneficial bacteria, reduction in potentially harmful bacteria and positive changes in markers of metabolic health.^160^ In another intervention, moderate consumption of red wine over a month increased alpha diversity and levels of some minor genera capable of metabolizing polyphenols.^161^ In contrast to these results, non-alcoholic beer resulted in more pronounced changes in gut microbiota diversity and composition than alcoholic beer, and enhancements in glucose tolerance which were not seen after the consumption of alcoholic beer, with these results suggesting that the presence of ethanol in alcoholic beer may blur potential favourable health effects of other nutrients.^162^ Future studies are needed to explore the impact of ethanol with/without other nutrients on the microbiome and musculoskeletal outcomes.

## Prebiotics and probiotics for the modulation of gut microbiome and musculoskeletal health in humans (adults)

Globally, the composition and metabolic activity of the gut microbiota could be manipulated through diet, antibiotics, probiotics (‘live microorganisms which, when administered in adequate amounts, confer a health benefit on the host’), prebiotics (non-digestible fermentable compounds that promote the growth or the activity of beneficial microbes), postbiotics (beneficial microbial metabolites such as SCFAs) or faecal microbiota transplantation. Since there are no available clinical studies on antibiotics, postbiotics and faecal microbiota transplantation with regards to osteoporosis, sarcopenia or osteoarthritis, in this review, we focus on interventions with prebiotics and probiotics supplemented in considerable quantities (e.g. soft chews, capsules, tablets, sprays and shakes) in patients with, or at risk of, these musculoskeletal diseases. Notably, most of the available studies have explored whether these strategies can prevent/protect against musculoskeletal diseases, while studies on the effects of prebiotics/probiotics as treatment strategies (either alone or as complementary to existing treatments) remain very limited.

### Intervention studies with prebiotics

Clinical studies evaluating the effects of prebiotics on outcomes related to musculoskeletal health and disease in adults are summarized in Table 1.

#### Calcium absorption

Administration of prebiotics has been shown to enhance calcium absorption in the majority of studies in adults including older individuals.^57,58,60–63,66^ Although overall beneficial effects have been observed with different types of prebiotics [inulin and oligofructose,^61,62^ soluble corn fibres,^66^ lactulose,^57^ galacto-oligosaccharides^58^ and fructo-oligosaccharides (FOS)^60^] provided at different doses (8–20 g), treatment effects are likely larger with greater doses and specific prebiotic types. For example, in postmenopausal women, an increase in calcium absorption was seen after a 9-day supply of lactulose at 10 g/day, but not after its provision at 5 g/day.^57^ Providing further evidence on dose-response effects, Jakeman *et al*.^66^ showed that the consumption of soluble corn fibres at doses of 10 and 20 g/day resulted in increases in skeletal calcium retention by 5% and 7%, respectively. Notably, most of the available studies reflect the short-term effects of prebiotics on calcium absorption (9 days to 8 weeks), while the long-term effects necessitate further investigation.

#### BMD and BTMs

Three randomized placebo-controlled trials have reported the effects of prebiotics on BMD. Two of them showed no significant effects of FOS^60^ or kefir-fermented milk^65^ on BMD in populations at increased risk for bone loss (postmenopausal women and older individuals). It is uncertain if these results reflect true absence of treatment effects or whether these interventions were too short (3–6 months) to capture small changes in bone mass. A study of longer duration (24 months) assessed bone turnover markers (BTMs) and BMD (by dual X-ray absorptiometry) changes in response to short-chain FOS plus calcium (CaFOS), calcium only or maltodextrin (placebo–control) in postmenopausal women.^64^ Treatment with CaFOS decreased serum C-telopeptide of type 1 collagen at 12 months and serum osteocalcin at 24 months to a larger extent than control. While these results indicated that CaFOS reduced bone turnover compared with other treatments, significant protection of total body BMD was only seen in the CaFOS group compared with the calcium-only group. Further, this study suggested that, among women with osteopenia, those who consumed CaFOS experienced lower reductions in spine BMD compared with those who were provided Ca only or placebo–control, with these results suggesting that this type of intervention may benefit more women at higher risk for osteoporosis. In accordance with the BTM findings of this study,^64^ several other investigations of shorter duration (2–12 weeks) have reported decreases in BTMs,^60,63,67^ although some others have shown increases^66^ or no effects.^59^ In summary, current evidence based on few investigations suggests modest benefits of prebiotics on skeletal health.

#### Sarcopenia/frailty-related outcomes

Prebiotic supplementation in frail individuals has been shown to increase the abundance of certain bacterial taxa with less pronounced effects on alpha and beta diversity.^163^ The evidence on the effects of prebiotics in sarcopenia/frailty-related outcomes is, however, limited. In a randomized controlled trial (RCT) exploring the effects of a mixture of inulin and FOS (*versus* placebo) in ambulatory elderly residing in nursing homes, the intervention group experienced significant improvements in handgrip strength and self-reported feeling of exhaustion after 13 weeks of supplementation, while the overall rate of frailty remained unchanged.^68^ By using a similar intervention (inulin + FOS *versus* placebo over a 13-week period), Theou *et al.*^69^ demonstrated a modest reduction in a 62-item frailty index in older individuals receiving the prebiotic mixture. Improvements in physical function, frailty degree, nutritional status and quality of life were also shown following a 12-week intervention with an oral nutritional supplementation (FOS and inulin, protein, Ca, Vitamin D) plus physical exercise in frail elderly (*versus* baseline, no control group); nevertheless, it was not possible to disentangle the contributions of the individual components of this multimodal intervention to the observed favourable changes.^164^ Collectively, the available studies suggest that prebiotics as stand-alone therapy or as complement to other treatments may positively influence some aspects of physical performance in frail individuals.

### Intervention studies with probiotics

Clinical studies evaluating the effects of probiotics on outcomes related to musculoskeletal health and disease in adults are summarized in Table 2.

**Table 2.** A summary of clinical studies evaluating the effects of probiotics on outcomes related to musculoskeletal health and disease in adults.

Study	Population (age, number)	Study design	Intervention	Control	Main findings
BTMs and BMD					
Jafarnejad *et al*.^165^	Postmenopausal women with osteopenia, age 50–72 years, *n* = 50	Double-blind, randomized, placebo-controlled design D = 6 months	seven probiotic bacteria species (1 capsule/day) + Ca 500 mg + vit D 200 UI/d	Placebo + Ca 500 mg + vit D 200 UI/d	↓ sCTX, BAP, PTH and TNFα in the probiotic group (*versus* control) NS differences in spine and total hip BMD between treatments
Lambert *et al*.^166^	Postmenopausal women with osteopenia, age 60–85 years, *n* = 78	Double-blind, placebo-controlled, randomized, parallel design, D = 12 months	60 mg isoflavone aglycones + probiotic lactic acid bacteria + Ca 1200 mg/day + Mg 550 mg/day + calcitriol 0.25 μg/day	Placebo + Ca 1200 mg/day + Mg 550 mg/day + calcitriol 0.25 μg/day	↓ sCTX in the probiotic group (*versus* control) ↓ BMD loss at the L2–L4 lumbar spine, femoral neck, and trochanter with probiotics (*versus* control)
Nilsson *et al*.^44^	Postmenopausal women with osteopenia, mean age 76 years, *n* = 90	Double-blind, placebo-controlled, randomized design D = 12 months	*Lactobacillus reuteri* 6475 (10^10^ CFU/d)	Maltodextrin powder	NS differences in BTM between treatments Lower ↓ in tibia total vBMD and trabecular bone volume fraction in the probiotic group (*versus* control)
Takimoto *et al*.^45^	Postmenopausal women with osteopenia, mean age 50–69 years, *n* = 61	Double-blind, placebo-controlled, randomized design D = 6 months	*Bacillus subtilis* (3.4 × 10^9^ CFU/d	Placebo	↓ uNTX and TRACP-5b (*p* = 0.052) in the probiotic group (*versus* control) at 12 weeks, but not at 24 weeks ↑ in total hip BMD in the probiotic group (*versus* control), but NS differences in lumbar spine BMD
Jansson *et al*.^167^	Early postmenopausal women, mean age 59 y, *n* = 249	Double-blind, placebo-controlled, randomized design D = 12 months	Three lactobacillus strains (*Lactobacillus paracasei* *DSM 13434*, *Lactobacillus plantarum* *DSM 15312*, and *L. plantarum DSM 15313* (10^10^ CFU/day)	Placebo	NS differences in BTMs between treatments ↓ LS-BMD loss, but ↑ loss in femoral neck BMD in the probiotic group (*versus* control)
Post-fracture recovery					
Lei *et al*.^168^	Older individuals with distal radius fracture, mean age 65 years, *n* = 381	double-blind, randomized, placebo-controlled design D = 6 months	skimmed milk containing *Lactobacillus casei Shirota* (6 × 10^9^ CFU/day)	Skimmed milk	DASH score, pain, CRPS score, wrist flexion and grip strength of patients on probiotics improved at a faster rate (*versus* control)
Osteoarthritis-related outcomes					
Lei *et al*.^169^	Patients with knee OA, mean age 67 years, *n* = 433	Double-blind, placebo-controlled, randomized design D = 6 months	Skimmed milk containing *L. casei Shirota* (1.2 × 10^10^ CFU/day)	Skimmed milk containing placebo	↓ serum hs-CRP levels, WOMAC and VAS scores in the probiotic group (*versus* control)
Lyu *et al*.^170^	Patients with knee OA, mean age 61–65 years, *n* = 80	Double-blind, placebo controlled, randomized design D = 12 weeks	TC1633 (*Streptococcus thermophilus*; 4 capsules of 5 × 10^8^ CFU/capsule per day)	Placebo	↓ sCTX-II and serum CRP in the probiotic group (*versus* control) NS differences in WOMAC between treatments

↑, increased; ↓, decreased; BMD, bone mineral density; BTMs, bone turnover markers; Ca, calcium; CFU, colony-forming unit; CRP, C-reactive protein; CRPS, complex regional pain syndrome; CTX, C-telopeptide of type 1 collagen; CTX-II, collagen type II C-telopeptide; D, duration; DAS, disease activity score; DAS28, Disease Activity Score of 28 Joints; DASH, disabilities of the arm; shoulder and hand; DPD, deoxypyridinoline cross-links; HAQ, health assessment questionnaire; hs-CRP, high-sensitivity C-reactive protein; *n*, number; LS, lumbar spine; Mg, magnesium; NS, non-significant; NTX, N-terminal telopeptide of type I collagen; OA, osteoarthritis; OC, osteocalcin; PTH, parathyroid hormone; s, serum; SJC, swollen joint count; TJC, tender joint count; TNF-α, tumor necrosis factor alpha; TRACP-5b, tartrate-resistant acid phosphatase isoform 5b; u, urinary; VAS, Visual Analogue Scale; v, volumetric; Vit, vitamin; WOMAC, Western Ontario and McMaster Universities osteoarthritis index.

#### Osteoporosis-related outcomes

Few RCTs have addressed the effects of probiotics on BTMs and bone mass.^44,45,165–167^ These have been conducted exclusively in postmenopausal women and have used several probiotic strains in variable doses over 6- to 12-month supplementation periods. Most of available studies (four out of five studies) support significantly attenuated bone loss in response to probiotic treatments.^44,45,165,166^ In one trial, probiotics co-administered with isoflavones mitigated BMD reductions at the lumbar spine, femoral neck and trochanter.^166^ Due to the study design, it was not, however, possible to tease out the individual effects of probiotics or isoflavones.^166^ The three other studies which demonstrated skeletal benefits, utilized distinct probiotic strains (*Lactobacillus reuteri*, *Bacillus subtilis* or a mixture of three lactobacillus strains) and overall showed reduced bone loss at the distal tibia,^44^ lumbar spine^167^ or the hip.^45^ It should be noted that the magnitude of the effects observed with probiotics (*versus* placebo at 12 months) is comparable with that achieved with calcium and/or vitamin D supplementation, but rather small compared with the magnitude of BMD improvements following treatment with osteoporosis medications.^13^ Mechanisms that may be driving the favourable skeletal influences of probiotics include reductions in bone resorption in some studies,^44,166^ modulations in gut microbiota^45^ and/or promotion of favourable oestrogen metabolite profiles.^166^ Although none of the aforementioned investigations has tested the effects of probiotics on fracture risk, a study conducted in older patients who sustained a fracture (distal radius) revealed that treatment with probiotics (skimmed milk containing *Lactobacillus casei Shirota*) significantly improved indicators of the fracture healing process including pain, disabilities of the arm, shoulder and hand, active range of motion and grip strength during the first 4 months after the injury compared with placebo.^168^

#### Osteoarthritis-related outcomes

We identified two probiotic interventions in patients with osteoarthritis. In one study, Western Ontario and McMaster Universities Arthritis index (WOMAC) and Visual Analogue Scale (VAS) scores improved and serum high-sensitivity C-reactive-protein (CRP) levels were significantly lower in patients administered *L. casei Shirota* compared with those receiving placebo.^169^ In another recent investigation, supplementation with another probiotic strain (*Streptococcus thermophiles versus* placebo) resulted in reductions in CRP and markers indicating degradation of type II collagen, but had no effect on WOMAC.^170^ Additional research is needed to enhance our understanding of the effects of probiotics in patients with different types of arthritis, while considering potential confounding factors, including age, sex, diet, medications and individual microbial signatures.

### Prebiotic and probiotic consumption: a holistic diet approach

In addition to interventional studies exploring the impact of prebiotic and probiotic administration in substantial amounts on bone, muscle and joint health, it is interesting to envisage whether foods and dietary patterns naturally rich in these ingredients could prevent or ameliorate musculoskeletal conditions. If effective, a food approach to manipulate the microbiome is worth considering for a number of reasons. It is affordable, promotes adequacy and variety and allows flexibility and personalized solutions. Furthermore, there is increasing interest in food matrices, which consider that whole foods/food groups are more than simply the sum of their components.^171^ Interestingly, patients with musculoskeletal conditions often feel ambivalently about drugs and pharmaceutically manufactured supplements and may be more receptive towards nutritional changes complementary to their therapeutic regimens.

To this end, several epidemiological studies have investigated the associations between diet and musculoskeletal outcomes or the association between dietary patterns and gut microbiome characteristics, but the three-way interplay has rarely been explored. Our group and others have shown that consumption of fermented dairy products (major dietary source of probiotics, but also of proteins, calcium and other micronutrients) were associated with higher BMD,^172^ favourable bone microstructure,^114^ lower bone loss^114^ and fracture rates;^173^ nevertheless, the potential links to changes in gut microbiota were not studied. Overall, the Mediterranean diet and other plant-based diets (characterized by high intakes of wholegrains, nuts, legumes, fruits and vegetables which are good sources of fibres, but also other nutrients with prebiotic-like or immunomodulatory effects such as polyphenols, antioxidants, monounsaturated fatty acids) have been associated with benefits for the prevention and/or management of musculoskeletal diseases,^174,175^ with the exception of vegan diets that exclude animal proteins and dairy products and have been associated with harmful effects on skeletal health.^176^ In separate investigations, such dietary patterns have also been linked to proliferation of beneficial bacteria, increases in SCFA synthesis, lower intestinal inflammation and limited bacterial translocation.^177,178^ A recent prospective investigation was the first to simultaneously assess the association between diet, microbiota patterns and frailty-related outcomes.^179^ They suggested that older individuals adhering to the Mediterranean diet had favourable changes in the gut microbiome, which were further associated with lower levels of inflammatory markers, greater walking speed and handgrip strength, and better cognitive function independent of major confounders (i.e. age, sex, body mass index).^179^ Future prospective studies and clinical trials are required to verify these findings and provide additional insights into the complex diets/microbiome/musculoskeletal health interactions.

## Conclusions and future perspectives

Over the past decade, experiments using different approaches and human investigations have provided exciting novel insights into the complex interplay between microbiome and treatment options in ageing-related musculoskeletal diseases. Nevertheless, current evidence is limited and often indirect. For example, our understanding of the interactions between the microbiome and drugs for osteoporosis treatment largely derives from animal experiments that have explored the contribution of the microbiome to key metabolic pathways involved in bone metabolism (i.e. PTH, oestrogen), rather than the microbial metabolism of the available drugs exploiting these pathways (teriparatide/abaloparatide, HRT/SERMs) or the effects of these drugs on microbiota characteristics. Furthermore, several studies have looked at the two-way interactions between lifestyle factors/drugs and musculoskeletal health outcomes or between lifestyle factors/drugs and microbiome; however, the three-way interactions have only rarely been assessed. Differences between conventional-raised, antibiotic-treated, and GF animals have provided preliminary data that the microbiome is involved in the metabolism of drugs in musculoskeletal diseases, nevertheless, well-controlled clinical trials are needed to explore how differences in microbiota composition affect outcomes in response to disease therapeutics. Further understanding of these aspects could aid the development of tools that consider patients’ gut microbiome and predict treatment responses, and drive advances in therapeutics based on, or targeted at, the gut microbiome, which may have the potential to improve therapeutics’ efficacy or reduce drug side effects. To this end, future studies are required to investigate the therapeutic use of prebiotic and probiotic supplementation as monotherapy or as adjunctive to conventional treatments for musculoskeletal diseases.
